# Effect of Native Oxide Film on Commercial Magnesium Alloys Substrates and Carbonate Conversion Coating Growth and Corrosion Resistance

**DOI:** 10.3390/ma7042534

**Published:** 2014-03-28

**Authors:** Sebastián Feliu, Alejandro Samaniego, Elkin Alejandro Bermudez, Amir Abdelsami El-Hadad, Irene Llorente, Juan Carlos Galván

**Affiliations:** 1 Centro Nacional de Investigaciones Metalúrgicas CSIC, Avda. Gregorio del Amo 8, Madrid 28040, Spain; E-Mails: samaniegoal@gmail.com (A.S.); irene@cenim.csic.es (I.L.); jcgalvan@cenim.csic.es (J.C.G.); 2 Departamento de Ciencias de los Materiales, Simon Bolivar University, Baruta, Caracas 1080-A, Venezuela; E-Mail: eabermudezm@gmail.com; 3 Physics Department, Faculty of Science, Al-Azhar University, Nasr City 11884, Cairo, Egypt; E-Mail: amirelhadad@cenim.csic.es

**Keywords:** magnesium, XPS, SEM, passivity, segregation

## Abstract

Possible relations between the native oxide film formed spontaneously on the AZ31 and AZ61 magnesium alloy substrates with different surface finish, the chemistry of the outer surface of the conversion coatings that grows after their subsequent immersion on saturated aqueous NaHCO_3_ solution treatment and the enhancement of corrosion resistance have been studied. The significant increase in the amount of aluminum and carbonate compounds on the surface of the conversion coating formed on the AZ61 substrate in polished condition seems to improve the corrosion resistance in low chloride ion concentration solutions. In contrast, the conversion coatings formed on the AZ31 substrates in polished condition has little effect on their protective properties compared to the respective as-received surface.

## 1. Introduction

Materials chosen for the study are Mg alloys which have aroused a great deal of scientific and technological interest over the past two decades. From a practical point of view, magnesium has the lowest density of all structural metals, making it highly attractive for use in the automotive, aerospace, IT and electronics industries where weight plays a decisive role. However, as magnesium is one of the most chemically active metals, insufficient resistance to atmospheric and aqueous corrosion sometimes limits its applications. Thus, it is desirable to have as complete as possible information on the factors that influence the corrosion of these materials.

Today’s eco-awareness coupled with the rapid growth of Mg alloys application in the automotive industry motivates the search for environmentally friendly treatments which enhance the corrosion resistance of magnesium alloy surfaces. Chemical conversion coatings stand out from other coating types that include anodising, electroplating, electroless plating, ion implantation, *etc*., owing to low cost and efficiency [1,2]. In general, no power or specific facilities are required to carry out conversion coating process, significantly reducing production cost [3]. Additionally, these chemical conversion coatings may be used as a pre-treatment to improve the adhesion or corrosion resistance of subsequent paint or organic layers on the surface of the magnesium alloy substrate [4]. Conversion treatments of Mg alloys in aqueous HCO_3_^−^/CO_3_^2−^ carbonate solutions [4–11], are becoming attractive procedures to reduce the corrosion rate of the substrate. Zuleta *et al*. [7] compared the different layers formed on the surface of pure magnesium from three chromium-free processes (anodizing and treatments with cerium salts and carbonates), and the calcium carbonate treatment appeared the most effective method to reduce the corrosion rate. Whereas the oxide layer formed in the anodizing process was a porous film made of MgO and some phosphate species compounds, the coatings obtained from a calcium carbonate treatment exhibited better corrosion protection due to formation of a compact, stable and adherent layer composed mainly of CaCO_3_ and MgO.

Although coating by chemical conversion in carbonic acid solution is a relatively clean method, it takes between 2 and 24 h to form a coating on Mg alloy substrates [4,6–11]. Therefore, some fundamental studies about the mechanisms involved in the growth of this type of coatings are essential in order to increase the kinetics of the process and to reduce the treatment time [11].

The properties of the thin oxide/hydroxide film formed on the surface of the magnesium alloys often determine the protective behavior of the conversion coatings. Assuming the hypothesis that the performance of the coating relies upon the chemistry of the oxide film that cover the alloy before the treatment, its characterization is of considerable importance. In the first stage of the conversion coatings growth process on magnesium alloys, there is dissolution of the native passive film accompanied by the formation of hydroxyl ions and pH rise [12,13]. Lin and Fang [14] proposed that after immersion in Ce(NO_3_)_3_, the air-formed magnesium oxide film immediately dissolves due to pH values below 8.5, which make it unstable. In our previous studies [15–18], we have observed that the properties of the thin oxide/hydroxide native oxide surface film (only a few nanometres thick) may affect the corrosion properties of magnesium alloys in the atmosphere [15,16] or in NaCl solution [17,18]. In a previous study [18], XPS (X-ray photoelectron spectroscopy) was used to characterize the differences in the oxide films formed on the surface of AZ31 and AZ61 alloys in as-received and freshly polished conditions. The findings revealed the presence of a significant fraction of the as-received alloy surface covered by islands of spinel (<3 nm in thickness) formed as a result of the manufacturing process. In immersion test in saline solution, during the initial stages of testing, considerable higher corrosion rates were obtained in the as-received specimens compared to the freshly polished ones. The degree of heterogeneity of the films present on as-received surfaces seemed to decrease their protective capacity compared to the more perfect and uniform oxide film formed on freshly polished surface. In the present work, we are trying to understand the influence of the protective properties of these surface films on the initial magnesium dissolution of AZ31 and AZ61 alloys when in contact with NaHCO_3_ saturated solution used in the treatment.

In a previous study [19], we commented that the conversion coating developed in aqueous NaHCO_3_ solution exerted a certain beneficial effect monitored by means of EIS (Electrochemical Impedance Spectroscopy) during the first hours or days of corrosion testing. However this effect tends to be quickly lost after a few days of corrosion testing indicating an only slightly durable protective action of the studied coating in presence of the highly aggressive 0.6 M NaCl solution.

It seems likely that the improvement factor of corrosion resistance for the NaHCO_3_ treated surfaces should be significantly better if the corrosion tests were carried out in milder corrosive environments. In this sense, it remains to be explored the effectiveness of the NaHCO_3_ conversion treatment in the case of Mg alloys exposed to other environments with lower chloride ion concentration than the 0.6 M NaCl solution, representative of the variable conditions of service that may be encountered in practice.

The objectives of this research are as follows:

1) To study the possible changes on the surface chemistry of the conversion coating formed on the AZ31 and AZ61 alloys treated with saturated aqueous NaHCO_3_ solution induced by the protective properties of the oxide film that forms spontaneously on the surface of magnesium alloys. In this sense, the research compares the behavior of substrates of the above alloys in the following two surface conditions: (a) specimens in as-received condition and (b) freshly polished specimens. The following nomenclature is used in the remainder of the paper to designate the four dual combinations tested: AZ31-O, AZ31-P, AZ61-O, and AZ61-P, where the letters O and P, that accompany the alloy type, denote: O = original surface condition (e.g., as-received condition); *p* = polished surface condition. The chemical nature and morphology of the surface after treatment is studied by XPS, scanning electron microscopy (SEM) and energy dispersive analysis of X-ray (EDX).
2) To contribute to a better understanding of the influence of surface chemistry of the conversion coatings developed in aqueous NaHCO_3_ solution and their corrosion resistance in saline solutions of different aggressiveness. The corrosion resistance of the treated surfaces is evaluated by means of EIS and hydrogen evolution measurement.

## 2. Results and Discussion

### 2.1. XPS Analysis of the Surface Chemistry of the AZ31 and AZ61 Substrates in Polished Surface Condition after NaHCO_3_ Treatment for Different Times

**Figure 1.**
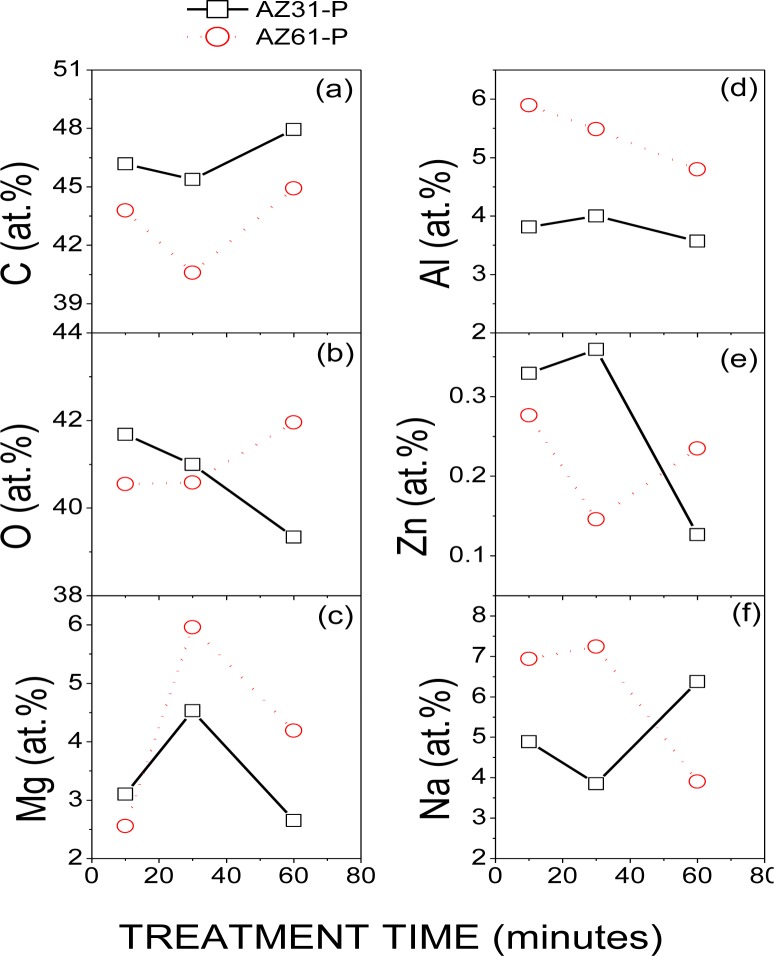
(**a**) Variation in the Carbon; (**b**) oxygen; (**c**) magnesium; (**d**) aluminum; (**e**) zinc and (**f**) sodium atomic percentages obtained by X-ray photoelectron spectroscopy (XPS) on the surface of the AZ31-P and AZ61-P substrates as a function of the treatment time in NaHCO_3_ saturated solution.

Figure 1 compares the evolution of the atomic percentages of C, O, Mg, Al, Zn and Na obtained by XPS on the surface conversion coatings formed on the AZ31-P and AZ61-P substrates with the treatment time in saturated NaHCO_3_ solution. No significant differences in these percentages were observed in the AZ31-P and AZ61-P specimens after different treatment times.

**Figure 2.**
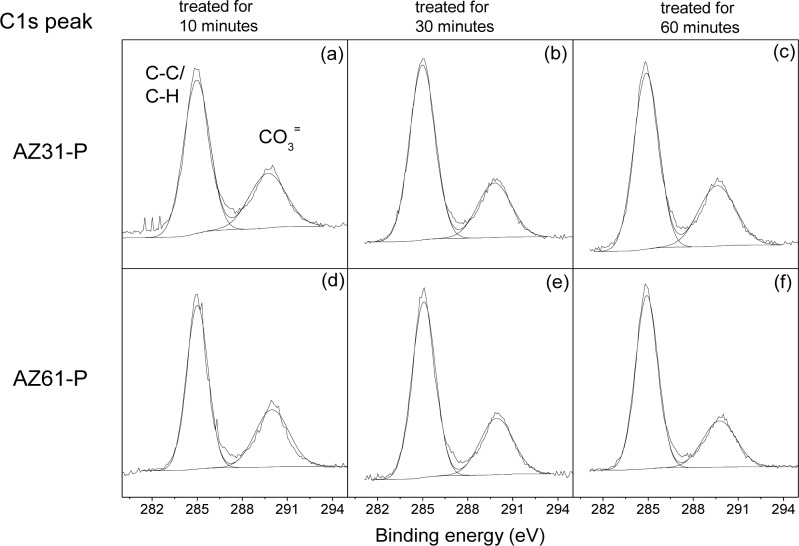
Variation in the C1s high resolution peak obtained by XPS on the surface of the AZ31-P and AZ61-P substrates as a function of the treatment time in NaHCO_3_ saturated solution.

Figure 2 compares the C1s high-resolution spectra obtained on the surface of the conversion coatings formed on the AZ31-P substrate treated for 10 min (a), for 30 min (b), for 60 min (c), with those obtained on the AZ61-P substrate treated for similar times (Figure 2d–f). The spectra may be fitted using two components at different binding energies. The first component is situated at approximately 285.0 eV, and is normally interpreted as carbon in the form of C–C/C–H groups; and a less intense component about 4.5–5.0 eV higher, which is associated with the presence of magnesium carbonate [20].

**Figure 3.**
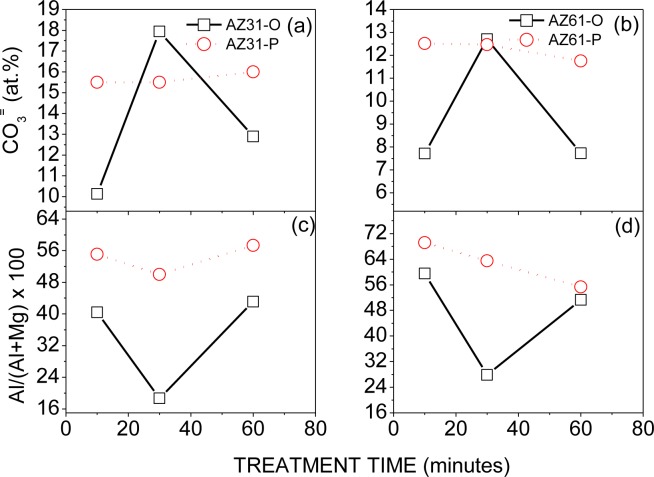
(**a**,**b**) Comparison of the atomic percentage of carbonate and (**c**,**d**) Al/(Al+Mg) × 100 obtained by XPS on the surface of the conversion coating as a function of the treatment time in NaHCO_3_ saturated solution and substrate surface conditions: (**a**,**c**) AZ31 alloy and (**b**,**d**) AZ61 alloy.

Figure 3 compares the variation in the atomic percentage of carbonate (a, b) and Al/(Al+Mg) × 100 atomic ratio (c,d) obtained by XPS on the surface of the AZ31 and AZ61 alloys as a function of the treatment time and substrate surface conditions. The atomic percentages of carbonate were obtained from the area of the second component used in the fitting of the C1s spectra (Figure 2) and the atomic percentages of C obtained by XPS on the surface of the AZ31-P and AZ61-P substrates after the treatment (Figure 1). In a previous study [19], we obtained the corresponding values for the AZ31-O and AZ61-O specimens. There is a significant increase in the carbonate content in the surface of the conversion coating formed on the AZ31-P and AZ61-P substrates after 10 and 60 min of treatment with respect to the AZ31-O and AZ61-O substrates for similar treatment times (Figure 3a,b). Likewise, in the surface of the conversion coating formed on the AZ31-P and AZ61-P substrates after 30 min of treatment there is a noteworthy increase in the Al/(Al+Mg) × 100 atomic ratio, approximately double with respect to the AZ31-O and AZ61-O substrates (Figure 3c,d).

The enrichment in carbonate and aluminum compounds observed by XPS as a function of the substrate surface condition and treatment time (Figure 3) may be related to the pH changes that occur directly above the metallic substrate surface during immersion in the NaHCO_3_ saturated solution. Using the XPS data, and taking into account into account the potential-pH diagrams of Al and Mg-water system [21], we can speculate on the influence of the native oxide surface film on the formation mechanisms of the conversion coating [22].

In the treatment solution, the overall reactions for the corrosion of magnesium can be listed as follows [23,24]:

$$\text{Mg}\to {\text{Mg}}^{2+}(\text{aq})+2{\text{e}}^{-}$$

$$2\;{\text{H}}_{2}\text{O}+2{\text{e}}^{-}\to {\text{H}}_{2}+2{\text{OH}}^{-}$$

$${\text{Mg}}^{2+}(\text{aq})+2{\text{OH}}^{-}(\text{aq})\to \text{Mg}{\left(\text{OH}\right)}_{2}(\text{s})$$

$$5\text{Mg}{\left(\text{OH}\right)}_{2}(\text{s})+4{\text{HCO}}_{3}{}^{-}(\text{aq})+4{\text{H}}^{+}\to {\text{Mg}}_{5}{\left({\text{CO}}_{3}\right)}_{4}{\left(\text{OH}\right)}_{2}\cdot 8{\text{H}}_{2}\text{O}$$

The carbonate contents determined by XPS on the surface of the AZ31-P and AZ61-P substrates after 10 min of treatment reach values of 50% and 60% higher than on AZ31-O and AZ61-O samples, respectively (Figure 3a,b). Taking into account the solubility products of the magnesium carbonate (6.82 × 10^−6^ [25]) and magnesium hydroxide in water (5.61 × 10^−12^ [25]) the solubility of hydroxide probes to be enormously higher than that of carbonate, so that this latter compound will preferably precipitate [26]. In the early stages of treatment, it is probable that the oxide film that forms spontaneously on the polished substrate surface, much more perfect and protective than the film on the as-received surface [17,18,27], contributes to a significant decrease in the magnesium dissolution process and the production of OH^−^ ions from the cathodic reaction decreasing the pH in the vicinity of the metal surface and allowing and favoring the stability and preferential formation of magnesium carbonate products rather than that of magnesium hydroxide.

After immersion times of 30 min one can observe a clear increase in the Al/(Al+Mg) ratio on the surface of the conversion coating formed on the AZ31-P and AZ61-P substrates compared to AZ31-O and AZ61-O specimens (Figure 3c,d). This surface enrichment in aluminum could be related to the stronger stability of the aluminum hydroxide compared to the magnesium hydroxide when the pH is close to neutral. It is reasonable to assume that the preferential deposition of carbonate species on the case of the conversion layer formed on the substrates in the polished surface condition after 10 min of treatment may be sufficiently protective to limit the substrate dissolution, decreasing the pH and resulting in an increase in the amount of aluminum hydroxides in the surface of the conversion coating.

For treatments whose time exceeds 30 min, no significant variations were observed by XPS in the content of carbonate or the Al/(Al+Mg) × 100 ratio on the surface of the conversion coating formed on the AZ31-P substrate (Figure 3a,c).

**Figure 4.**
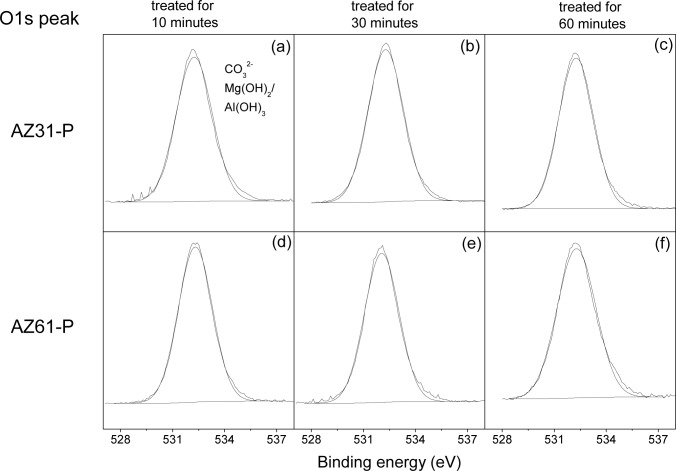
Variation in the O1s high resolution peak obtained by XPS on the surface of the AZ31-P and AZ61-P substrates as a function of the treatment time in NaHCO_3_ saturated solution.

Figure 4 compares the high resolution O1s spectra obtained on the surface of the conversion coatings formed on the AZ31-P substrate treated for 10 min (a), 30 min (b) and 60 min (c), with those obtained on the AZ61-P substrate treated for similar times (Figure 4d–f). The spectra obtained are fairly similar, containing one single component at a binding energy of 532.2 eV associated with the presence of magnesium carbonate or magnesium hydroxide form [28] and/or Al(OH)_3_ [29].

**Figure 5.**
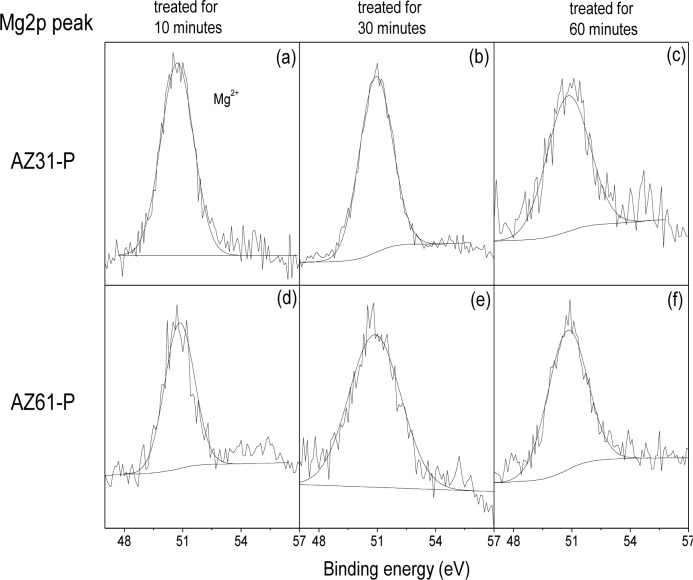
Variation in the Mg2p high resolution peak obtained by XPS on the surface of the AZ31-P and AZ61-P substrates as a function of the treatment time in NaHCO_3_ saturated solution.

Figure 5 compares the high resolution spectra Mg2p peak obtained on the surface of the conversion coatings formed on the AZ31-P substrate treated for 10 min (a), for 30 min (b), for 60 min (c), with those obtained on the AZ61-P substrate treated for similar times (Figure 5d–f). The spectra obtained contain one single component at a binding energy of 50.8 eV associated with the presence of magnesium in the form of magnesium hydroxide/carbonate [30].

**Figure 6.**
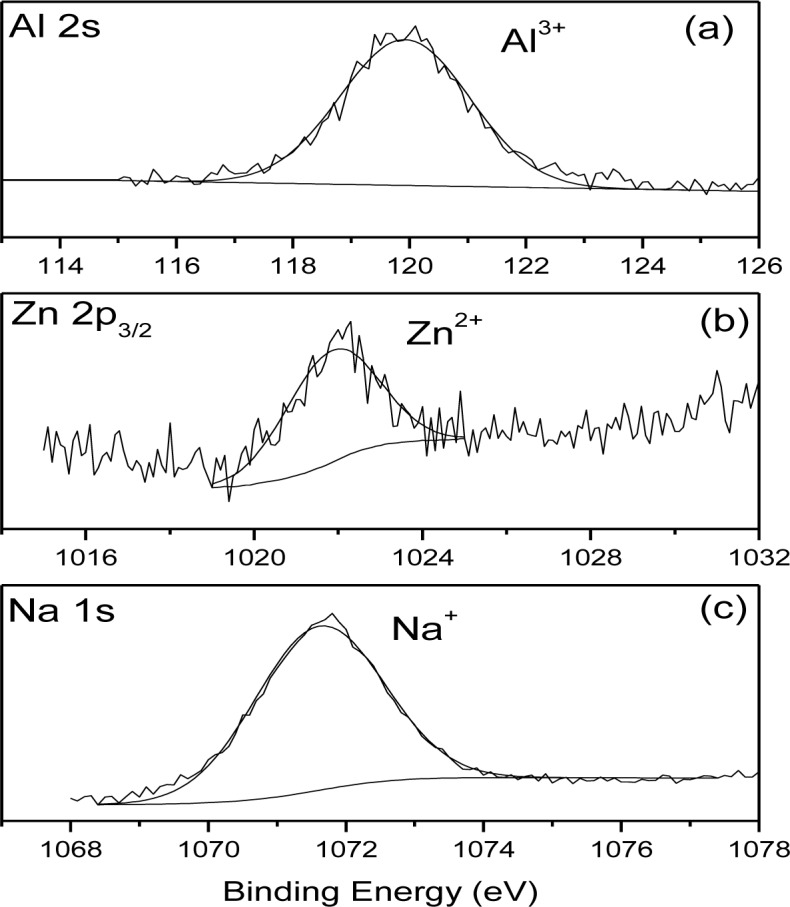
(**a**) High resolution Al 2s; (**b**) Zn 2p_3/2_ and (**c**) Na 1s XPS peaks obtained on the surface of the AZ31-P substrate after 10 min of treatment in NaHCO_3_ saturated solution.

Figure 6 shows the Al2s (a), Zn 2p_3/2_ (b) and Na 1s (c) XPS high resolution spectra obtained on the surface of the AZ31-P substrate after 10 min of treatment. These spectra are representative of the similar Al 2s, Zn 2p_3/2_ and Na1s spectra obtained on the surface of the AZ31-P and AZ61-P substrates after other treatment times. In the Al 2 s spectrum (Figure 6a) there is a component at 120.0 eV characteristic of aluminum in ionic state (Al^3+^ type). The Zn2p_3/2_ high resolution spectrum (Figure 6b) may be fitted to one component with a binding energy of 1022.0 eV associated with the presence of Zn^2+^. Finally, the Na1s spectrum (Figure 6c) may be fitted to one component at 1071.7 eV associated with the presence of sodium ions (Na^+^).

### 2.2. Microstructure of the Conversion Coating Formed on the AZ31 and AZ61 Alloy Substrates in Polished Condition after NaHCO_3_ Treatment for Different times

**Figure 7.**
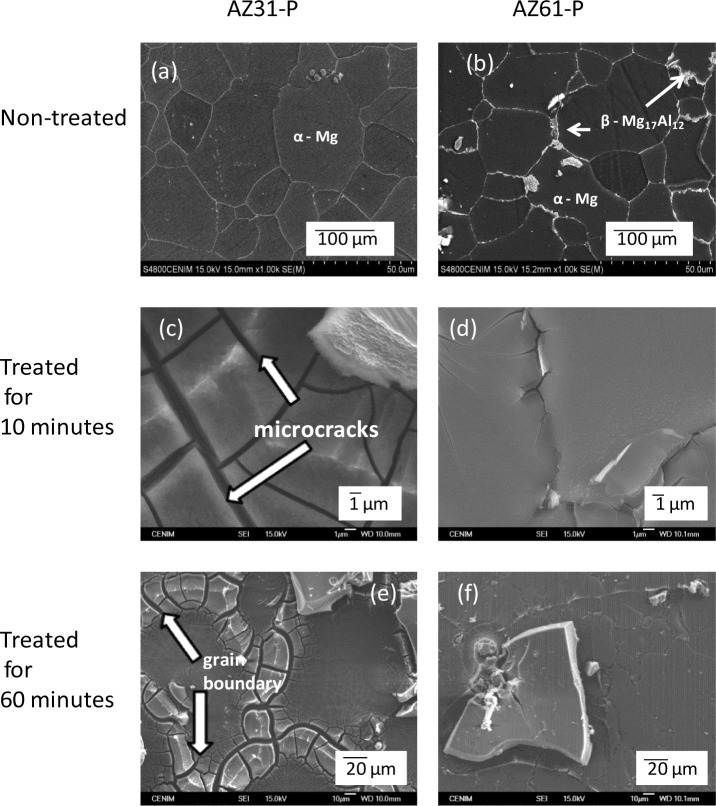
(**a**,**c**,**e**) SEM surface morphologies for AZ31-P and (**b**,**d**,**f**) AZ61-P substrates (**a**,**b**) non-treated (**c**,**d**) treated for 10 min and (**e**,**f**) treated for 60 min in saturated NaHCO_3_ solution, respectively.

Figure 7 compares the surface microstructures for the non-treated AZ31-P and AZ61-P substrates and treated in NaHCO_3_ saturated solution for 10 and 60 min. It is important to note that, from the early stages of the treatment, micro-cracks appear on the conversion coating formed on the AZ31-P substrate (Figure 7c). In Figure 7e, which represents the visual appearance of the surface of the AZ31-P substrate treated for 60 min, one can see that the layer is not uniform, and that there are areas without apparent cracks coexistent with other areas with visible cracks, particularly located at grain boundaries (as marked by arrowheads in Figure 7e). The growth of porous, non-uniform coatings with cracks and particularly poor coverage at the grain boundaries during chemical conversion treatment of aluminum alloys has been reported by Lunder *et al*. [31] and would appear to be a result of the galvanic coupling between the grain boundary/matrix when the grain boundaries apparently became preferred anodic sites and the matrix acts as a cathode. In the present work, this effect seems to be reflected in the visual appearance of the surface of the AZ31-P alloy treated for 60 min (Figure 7e). The microstructure of the non-treated AZ31 alloy is formed practically by an α matrix with Al in solid solution surrounded by grain boundary free of precipitates of β phase (Figure 7a). In the treatment solution, the grain boundaries selectively react because they are more active than grain bulk, motivating the growth of a very defective and heterogeneous layer of conversion coating in this alloy.

In contrast with the conversion coating formed on the AZ31-P substrate (Figure 7c), when the AZ61-P substrate is treated for 10 min (Figure 7d) the dark film looks smoother, more uniform and there are no visible micro-cracks. After 60 min of treatment, no similar defects to the treated AZ31-P substrate (Figure 7e) were observed on the surface of the conversion layer formed on the AZ61-P substrate (Figure 7f). As shown in Figure 7b, the aluminum is distributed, forming part of the chemical composition of the β-phase precipitates along the grain boundary of the AZ61 alloy. The notable difference between the electro-chemical potentials of the β and α phases suggests that during the conversion treatment the anodic reaction is supported by hydrogen evolution at the cathodic β phase contributing significantly to increase the pH in the vicinity of this phase and preferential precipitation of carbonates has occurred on the top of the β phase. It is likely that the quick blockage of the β phase in the AZ61 alloy increases the barrier effect of the grain boundary, leaves the metal surface in a less active state, motivating the growth of a more perfect, uniform, protective conversion layer than that which results on the AZ31 alloy. In a previous study [19], similar differences were observed on the conversion coatings formed on the AZ31 and AZ61 substrates in as-received surface condition.

**Figure 8.**
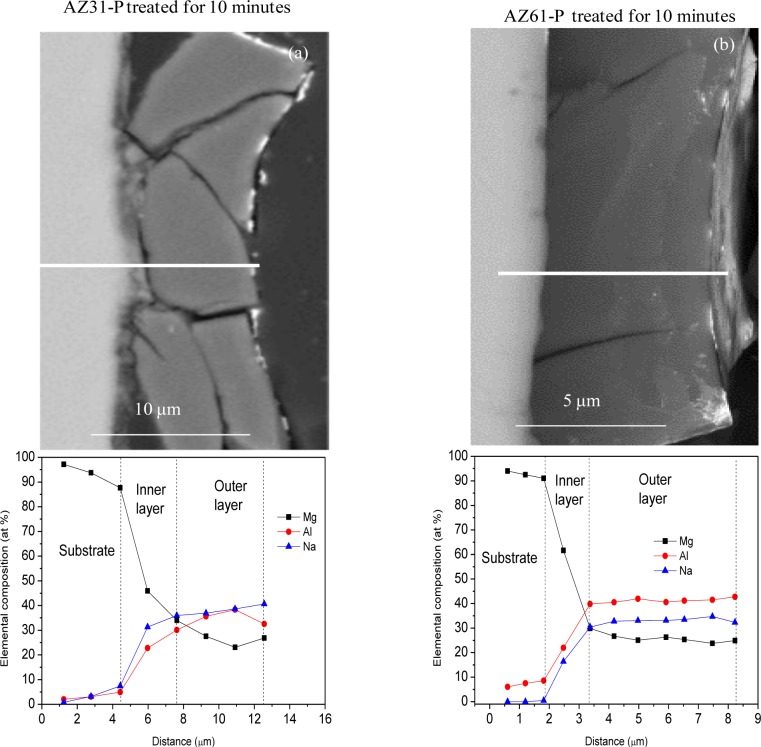
(**a**) SEM morphology and energy dispersive analysis of X-ray (EDX) quantitative analysis for the cross-section of the conversion coating formed on the AZ31-P; and (**b**) AZ61-P substrates after 10 min of treatment in NaHCO_3_ saturated solution.

Figure 8 compares SEM images and EDX quantitative analysis for the cross-section of the coating formed on the AZ31-P (a) and AZ61-P substrates (b) treated for 10 min in saturated NaHCO_3_ solution. Attention is drawn to the presence of significant cracks or discontinuities throughout the thickness of the conversion coating on the AZ31-P substrate (Figure 8a). The conversion coating formed on the AZ61-P substrate (Figure 8b) appears to be much more uniform and compact, and probably protective, than that formed on the AZ31-P substrate. The EDX analysis of the AZ31-P substrate treated for 10 min (Figure 8a) shows how the magnesium, aluminum and sodium contents observed on the outer layers of the conversion coating remain stable. In contrast to the presence of a fairly homogeneous layer noted above, on the inner layers of the conversion coating there is a notable decrease in aluminum and sodium contents and an increase in magnesium content toward the substrate. The composition of the conversion coating formed on the AZ61-P substrate treated for 10 min (Figure 8b) seems to be fairly similar to that formed on the AZ31 alloy (Figure 8a).

### 2.3. Protective Properties of NaHCO_3_ Treatment

#### 2.3.1. Hydrogen Evolution Measurements as a Function of Substrate Surface Condition, Immersion Time and Chloride Ion Concentration

**Figure 9.**
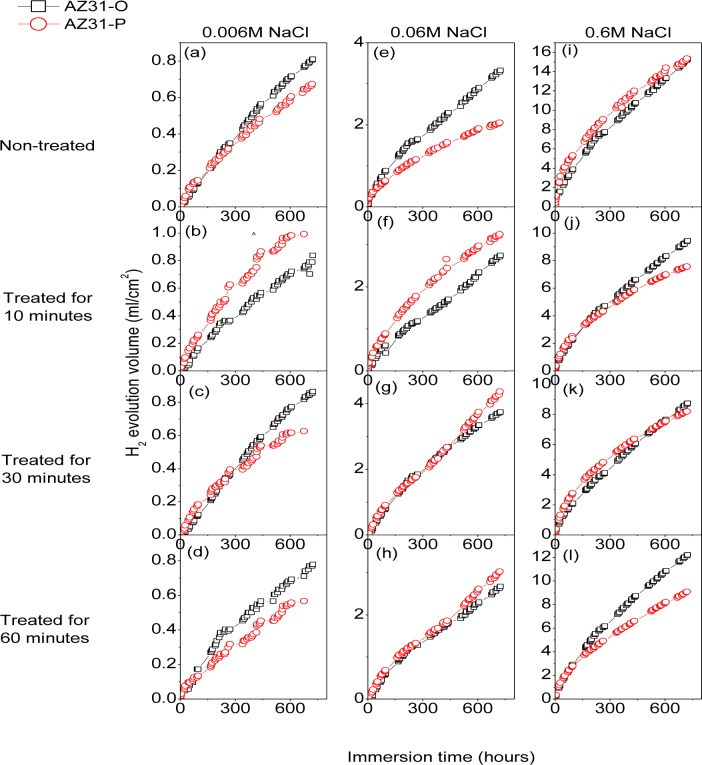
Comparison of the hydrogen evolution with different treatment times for AZ31-O and AZ31-P substrates during 700 h of immersion in saline solutions with different chloride ion concentrations.

Figure 9 compares the hydrogen evolution *versus* time curves (direct measure of the corrosion rate) for the AZ31-P substrate non-treated and after 10, 30 and 60 min of NaHCO_3_ treatment and those corresponding to the AZ31-O substrate during immersion in 0.006 M NaCl (a–d), 0.06 M NaCl (e–h) and 0.6 M NaCl (i–l) for 700 h. No significant differences were observed in these curves for the AZ31-P substrate after different treatment times compared to the AZ31-O substrate, regardless of the chloride concentrations of the solution (Figure 9).

**Figure 10.**
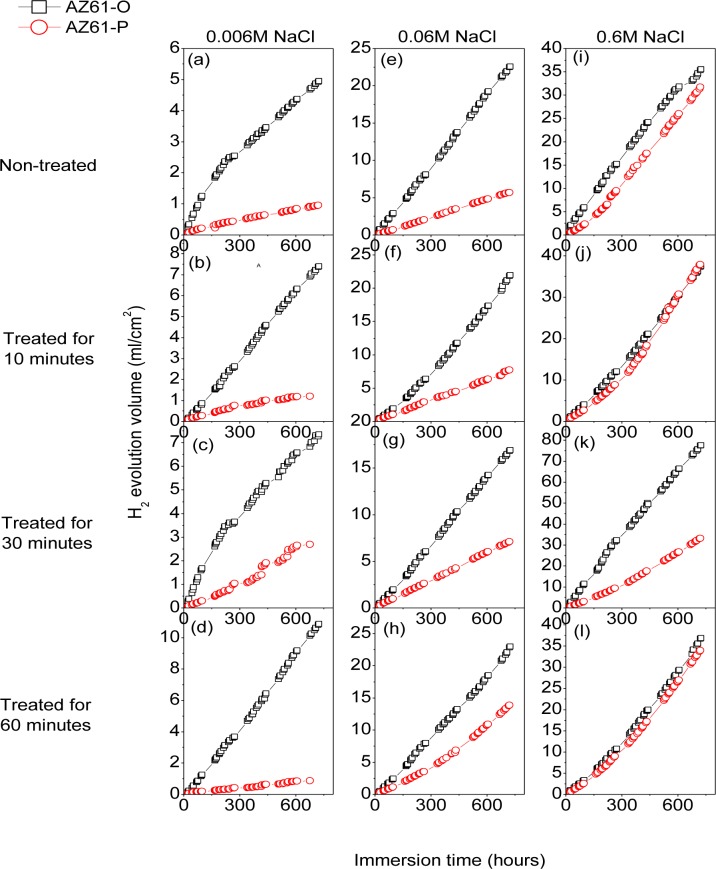
Comparison of the hydrogen evolution with different treatment times for AZ61-O and AZ61-P substrates during 700 h of immersion in saline solutions with different chloride ion concentrations.

Figure 10 compares the hydrogen evolution *versus* time curves for the AZ61-P substrate non-treated and after 10, 30 and 60 min of NaHCO_3_ treatment and those corresponding to the AZ61-O substrate during immersion in 0.006 M NaCl (a–d), 0.06 M NaCl (e–h) and 0.6 M NaCl (i–l) for 700 h. In contrast with the AZ31 alloy, significantly lower hydrogen evolution data were observed in the AZ61-P specimens during immersion in 0.006 M (Figure 10a–d) and 0.06 M NaCl (Figure 10e–h) than those corresponding to the AZ61-O substrate. Also, lower values of hydrogen volume were measured in the AZ61-P substrate treated for 30 min compared to those of the AZ61-O substrate during immersion in 0.6 M NaCl (Figure 10k).

**Figure 11.**
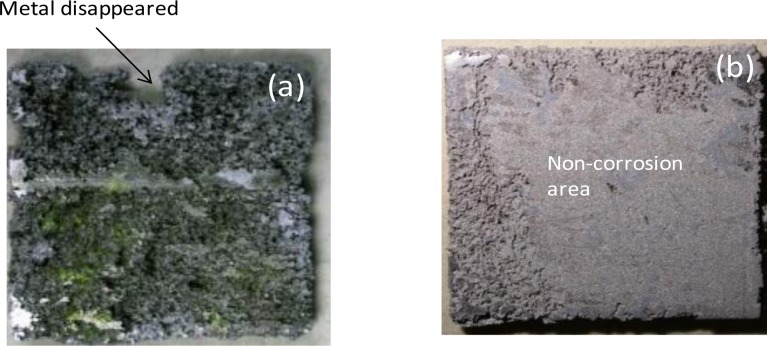
Comparison of the macroscopic surface appearance of the (**a**) coated AZ61-O treated for 30 min and (**b**) AZ61-P treated for 30 min, after 700 h of immersion in NaCl 0.6 M and after corrosion product removal.

Figure 11 compares the macroscopic surface appearance of the corroded AZ61-O and AZ61-P substrates treated for 30 min, after 700 h of immersion in NaCl 0.6 M and after corrosion product removal. In the sample AZ61-O one can observe uniform attack on large areas of the exposed surface and it is worth noting that there are areas in which the metal has disappeared, mainly around the edges (Figure 11a). However, in the AZ61-P specimen (Figure 11b), no metal disappeared from the borders. Likewise, it is interesting to note the predominance of non-corroded areas which appear to occupy more than 50% of the exposed surface (Figure 11b). In general, there is a qualitative agreement between the largest fraction of the corrosion area of the samples (Figure 11) and the hydrogen evolution data (Figure 10k).

#### 2.3.2. Electrochemical Impedance Measurements as a Function of Immersion Time in NaCl 0.006 M Solution

**Figure 12.**
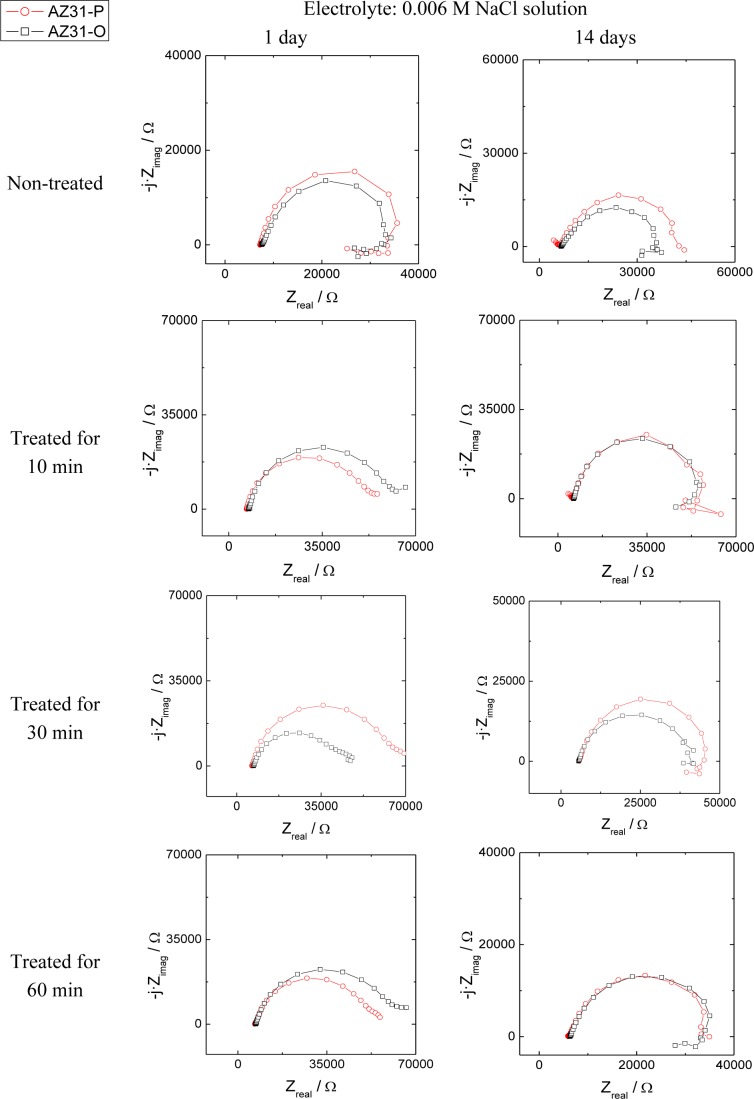
Comparison of the Nyquist plot with different treatment times for AZ31-O and AZ31-P substrates with immersion time in 0.006 M NaCl.

**Figure 13.**
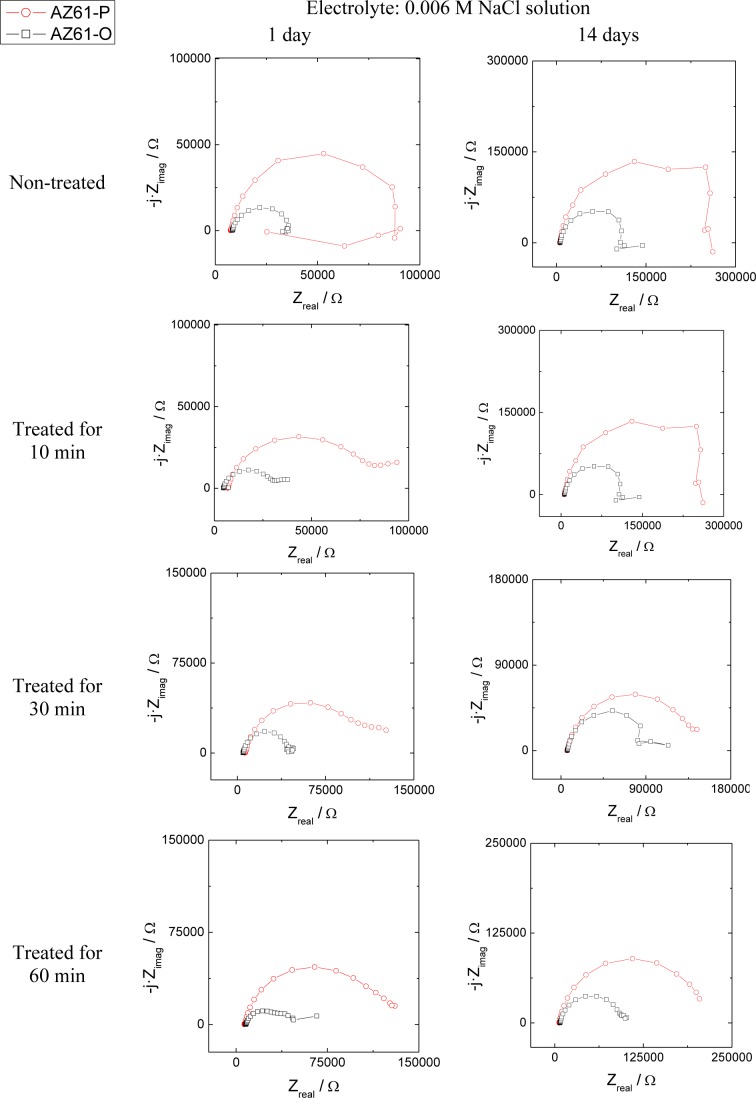
Comparison of the Nyquist plot with different treatment times for AZ61-O and AZ61-P substrates with immersion time in 0.006 M NaCl.

The evolution of the corrosion process on the treated AZ31 and AZ61 alloys as a function of the treatment time and substrate surface conditions has been monitored by means of impedance measurements during immersion in 0.006 M NaCl solution. Nyquist diagrams (Figures 12 and 13) show apparently one single capacitative loop at high frequencies (HF) during the different stages of testing. In some specimens, an inductive loop at low frequencies (LF) tends to become more or less patent (Figures 12 and 13).

**Figure 14.**
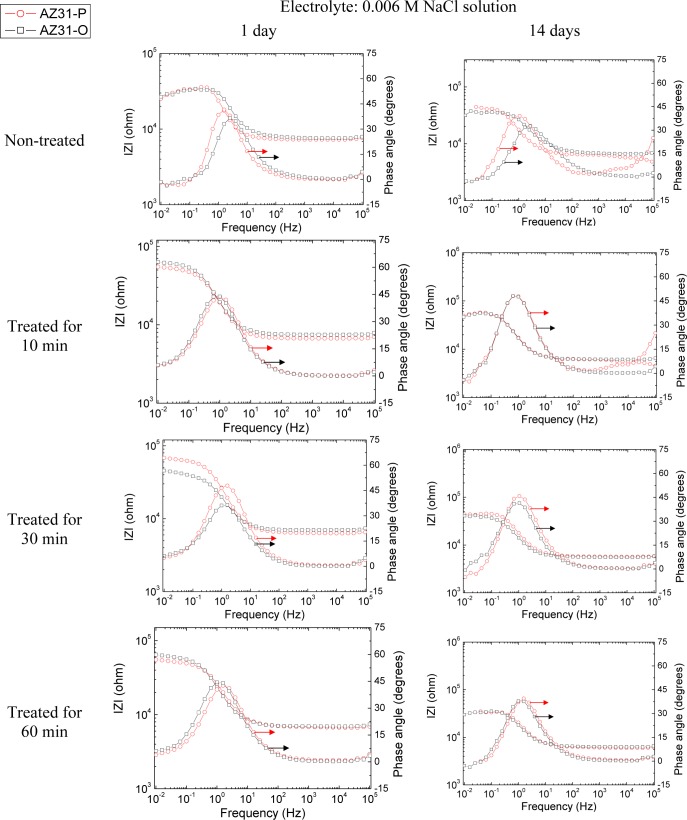
Comparison of the Bode plot with different treatment times for AZ31-O and AZ31-P substrates with immersion time in 0.006 M NaCl.

**Figure 15.**
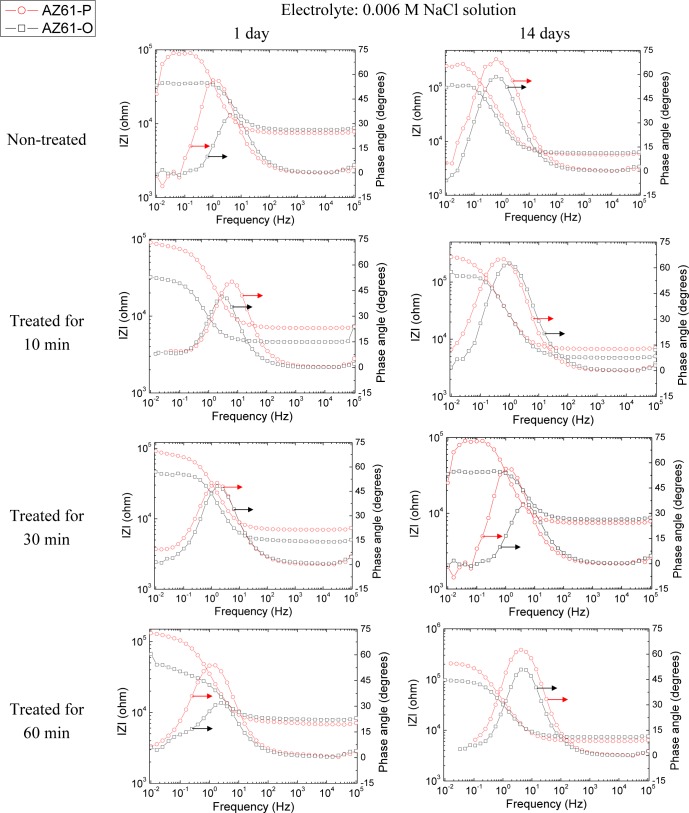
Comparison of the Bode plot with different treatment times for AZ61-O and AZ61-P substrates with immersion time in 0.006 M NaCl.

Representative impedance spectra of the tested specimens in terms of Bode plots are shown in Figures 14 and 15. They seem to show apparently one time constant.

Many studies in the literature [32–34], support the use of the charge transfer resistance (*R*_CT_), deduced from HF capacitive loop, to obtain information on the corrosion rate of magnesium alloys. It is normal to associate the diameter of this capacitive loop with the charge transfer resistance of the corrosion process [31,32,35], which is inversely related to the corrosion current (*i*_corr_) through the well known Stern-Geary Equation [36]:

$$i_{\text{coor}}=\frac{B}{R_{\text{CT}}}$$ (1)

The *R*_CT_ values, corresponding to the HF capacitive loop, were derived from the impedance data in the range of 10^5^ to 10 Hz. With the help of Zview software fitting method [37] the results were adequately fitted using an equivalent circuit formed by the charge transfer resistance and a constant phase element in parallel.

**Figure 16.**
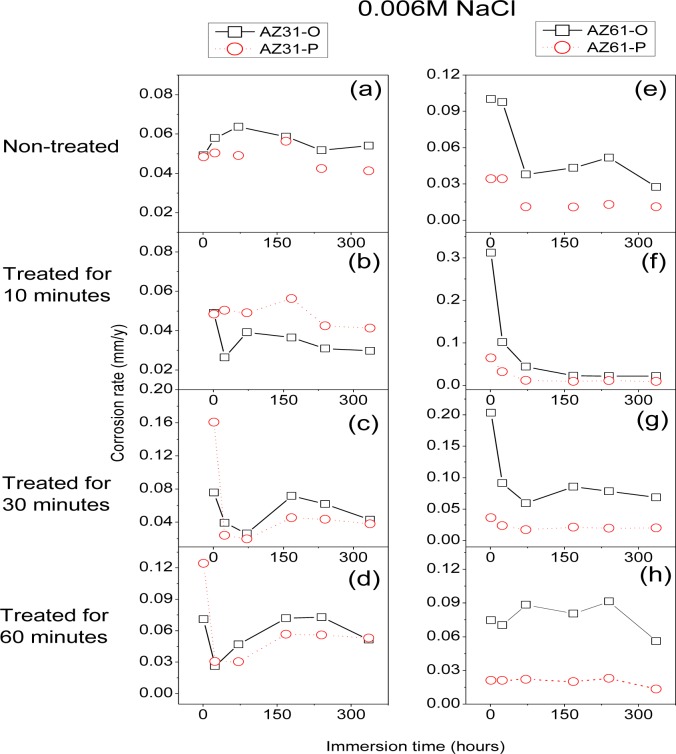
Variation in corrosion rate values as a function of alloy type and surface condition over 14 days immersion in 0.006 M NaCl.

Corrosion rates in Figure 16 were obtained from *R*_CT_ values by means of Equation 1, in which the constant of proportionality B (about 65 mV for the AZ31 alloy and 120 mV for the AZ61 alloy) was empirically determined by the correlation between electrochemical and gravimetric measurements. Corrosion rates in mA/cm^2^ were converted to corrosion rates (mm/y) by applying Faraday law. No significant differences in these values were observed in the AZ31-P substrate after different treatment times compared with those corresponding to the AZ31-O substrate (Figure 16a–d). In contrast with the AZ31 alloy, significantly lower corrosion rate values than the AZ61-O substrate after different treatment times were observed in those corresponding to the AZ61-P substrate (Figure 16e–h). It is interesting to note that similar trends regarding the corrosion behavior are deduced from these electrochemical values as from the hydrogen evolution ones (Figure 9a–d and Figure 10a–d).

Regarding the inductive loop observed at LF, several processes can induce this behavior in the corrosion of magnesium and its alloys. In the literature [38–42] it is mainly attributed to the relaxation of adsorbed species, such as Mg^2+^ or MgOH^+^ on the electrode surface and, also, to the possible dissolution of partially protective surface films although it is not always easy to find a definitive explanation to these inductive loops [38].

Because the inductive loops are not directly related to the rate of corrosion, their interpretation is considered immaterial as to provide kinetic information about the corrosion process.

As the results of the immersion tests, the conversion coatings formed on the AZ31-P substrate in polished condition do not reveal any particularly significant difference in corrosion resistance compared to those formed on the AZ31-O (Figure 9 and Figure 16a–d). As shown in Figure 8a, the conversion coating formed on the AZ31-P substrate after 10 min of treatment is thick and some visible and continuous cracks can be observed from the outermost surface of the coating to the substrate. This is an important feature suggesting that the coating is permeable to solution. When AZ31 alloy with the conversion coating is immersed in saline solution, the electrolyte could easily penetrate through the cracks of surface film to result in its corrosion resistance reduction [2].

Compared with the AZ31 alloy, (Figure 8a), the conversion coating formed on the AZ61 alloy is far more perfect and uniform (Figure 8b). Probably the nature and stability of the conversion coating formed on the AZ61 alloy play a role in the magnesium alloy corrosion process. Thus, it is reasonable to suppose that differences in the surface chemistry of the conversion coatings may exercise some influence on their stability in the immersion tests carried out in NaCl solutions.

Electrochemical impedance results (Figure 16) and hydrogen evolution *versus* time curves (Figure 10a–d) have provided information on the effect of experimental variables on the corrosion resistance of the specimens tested. If, as a reference, we use the data obtained from the coated AZ61-O substrates treated for 10 and 60 min during the immersion test in 0.006 M NaCl, it is clear the trend of the corresponding coated AZ61-P substrates to present lower corrosion rate (Figure 16f–h) and hydrogen evolution values (Figure10b–d) for the same immersion times. Similar trends are observed with the 0.06 M NaCl solution (Figure 10f–h). Comparing these results with the chemical composition obtained by XPS on the surface of the conversion layers resulting from the treatment, one clearly see a tendency towards a decrease in the hydrogen evolved as the carbonate content increase (Figure 3b). This correspondence suggests that the enrichment in carbonate species in the conversion coating probably controls the corrosion process in posterior immersion in saline solutions of weak (0.006 M NaCl) or medium (0.06 M NaCl) aggressiveness. In the literature [24,43], it is reported that magnesium hydroxyl carbonates products are non-conducting and could not serve as a substrate for the cathodic reaction. But, it is possible that they slow down the corrosion rate by blocking the anodic areas due to the decreased regions of free ion motion [23]. In this work, the replacing of the Mg(OH)_2_ with the more protective magnesium carbonate products on the conversion coating formed in the substrate in polished surface condition would increase their protective properties. Chloride-induced corrosion is thus retarded by this product to give a continuous coverage of the metallic surface [23].

In the immersion test in 0.6 M NaCl solution, the hydrogen volume evolved values for the coated AZ61-P and AZ61-O substrates treated for 10 (Figure 10j) and 60 minutes (Figure 10l) tend to be equal. In such aggressive medium, the enrichment in carbonate in the conversion coating formed on the substrate in as-polished condition does not result in a significant change in its corrosion resistance. These data may suggest that the chloride ion concentration in 0.6 M NaCl is aggressive enough to penetrate easily the carbonate film and significantly damage their blocking effect. A similar effect from chloride ion concentration has been observed by Liu *et al*. [44] in the corrosion behavior of AM60 magnesium alloy during immersion tests in aqueous solution.

In the immersion test in the 0.006, 0.06 and 0.6 M NaCl, the AZ61-P substrate treated for 30 min shows lower volumes of hydrogen evolved (Figure 10c,g,k) and corrosion rate (Figure 16g) values than those obtained in the corresponding AZ61-O for the same immersion times. XPS analysis has revealed as a distinctive characteristic of these specimens a higher Al/(Al+Mg) ratio on the surface of the conversion coating that forms on the AZ61-P substrate than the one observed on the AZ61-O substrate (Figure 3d). This correspondence suggests that the enrichment of the aluminum oxide/hydroxide on the surface of the conversion layer as a result of the treatment probably controls the corrosion process in posterior immersion in 0.6 M NaCl. Many studies mention the beneficial effect of Al [16,45–50] which may become the essential factor in determining the passivity of the surface, improving the resistance to local breakdown of the oxide and reducing the chance of chloride penetrating as far as the surface. In the literature [51], it is presumed that Al_2_O_3_ component forms a continuous skeletal structure in an amorphous matrix, so that the film properties become predominantly determined by the protective properties of Al_2_O_3_ very superior to that of Mg(OH)_2_. The presented results are consistent with our previous study [19] where we observed that the significant increase in the amount of aluminum oxides and hydroxides observed on the surface of the conversion coating of the AZ61 substrate in as-received condition after 10 or 60 min of treatment (about 30% higher Al atomic contents) seemed to improve the corrosion resistance in 0.6 M NaCl.

## 3. Experimental Section

The chemical compositions of the tested magnesium alloys, AZ31 and AZ61, are listed in Table 1. They were fabricated in wrought condition and supplied in plates of 3 mm thickness by Magnesium Elecktron Ltd, Manchester, UK.

This research compares the behavior of specimens of the above alloys in the following surface conditions:

Specimens in the as-received condition, where the untreated surfaces were only cleaned with distilled water and dried with hot air.

Freshly polished specimens were dry ground through successive grades of silicon carbide abrasive paper, from P600 to P2000, followed by finishing with 3 and 1 μm diamond paste, cleaned in distilled water and dried with hot air. Due to the high affinity of magnesium to the ambient atmosphere, it was attempted to keep the exposure time to the atmosphere before their subsequent immersion on saturated aqueous NaHCO_3_ solution to a minimum, around few hours.

The carbonate coating was formed chemically at room temperature, based on previous work by Al-Abdullat *et al*. [52]. The substrates were immersed into 4 L of aqueous NaHCO_3_ solution at a concentration of 9 mass% or saturation. The surface treatment was allowed to proceed for a given time at laboratory temperature followed by rinsing with distilled water and then air dried.

Photoelectron spectra were recorded using a Fisons MT500 spectrometer equipped with a hemispherical electron analyzer (CLAM 2) and an Mg Kα X-ray source operated at 300 W. The samples were fixed on small flat discs on a *XYZ* manipulator placed in the analysis chamber. The residual pressure in this ion-pumped analysis chamber was maintained below 10^−8^ torr during data acquisition. The spectra were collected for 20–90 min depending on the peak intensities, at a pass energy of 20 eV, which is typical of high-resolution conditions. The intensities were estimated by calculating the area under each peak after smoothing and subtraction of the S-shaped background and fitting the experimental curve to a combination of Lorentzian and Gaussian lines of variable proportions. Although specimen charging was observed, it was possible to determine accurate binding energies (BEs) by referencing to the adventitious C1s peak at 285.0 eV. The atomic ratios were calculated from the peak intensity ratios and the reported atomic sensitivity factors [53]. The measurements were performed at take-off angles of 45° with respect to the sample surface. The sampled areas were 1 mm × 1 mm. C1s, O1s, Mg2p, Al2s, Zn2p and Na1s high resolution XPS spectra were obtained on the non-sputtered surface of the conversion coating.

The tested specimens were examined by scanning electron microscopy (SEM) using a JEOL JXA 840A unit operating with Rontec EDR288 software for EDX spectra acquisition and image digitalisation.

For the hydrogen evolution determinations, the corrosion of magnesium alloys during solution immersion was estimated by determining the volume of hydrogen evolved during the corrosion process. Samples for hydrogen collection were cut into square coupons with dimensions of 2 cm × 2 cm× 0.3 cm and vertically immersed in 700 mL of quiescent 0.006 M NaCl, 0.06 M NaCl and 0.6 M NaCl for 28 days in a beaker open to laboratory air at 25 ± 2 °C. The entire specimen surface was exposed to the electrolyte. Evolved hydrogen was collected in a burette above an inverted funnel placed centrally above specimen. All these experiments were run simultaneously and each sample was subjected to essentially the same temperature and exposure history. The experimental difficulties and limitations of such test were recently documented [54].

The morphology of the attack on the corroded surface was examined at low magnification and a camera was used to take the photographic images. Once the test was finished, the corroded specimens were stripped in a solution of 200 g/L CrO_3_ and 10 g/L AgNO_3_ at room temperature to eliminate the corrosive products remaining on the surface, then rinsed with isopropyl alcohol and dried in hot air in order to study the corrosion morphology.

Electrochemical impedance measurements were conducted in 0.006 M NaCl after 1 h, 1 day, 3, 7, 10 and 14 days of exposure at room temperature (25 °C). An AUTOLAB potentiostat, model PGSTAT30, with frequency response analyzer (FRA) software was used. The frequency ranged from 100 kHz to 1 mHz with 5 points/decade, whereas the amplitude of the sinusoidal potential signal was 10 mV with respect to the open circuit potential in a steady state. The electrochemical system used for this purpose included graphite electrode (counter electrode), saturated Ag/AgCl electrode (reference electrode) and metal sheet (working electrode).

## 4. Conclusions

(1) XPS analysis has been used to quantify and compare the chemical changes on the surface of the conversion coatings formed on the AZ31 and AZ61 alloys treated with saturated aqueous NaHCO_3_ solution induced by the initial substrate surface condition and treatment times. Attention is drawn to the considerable surface enrichment in magnesium carbonates and aluminum hydroxide in the conversion coatings formed as a result of polishing the substrate. Close to two times higher amounts of carbonate of magnesium and aluminum hydroxides have been measured in the surface of the coatings formed on the substrates in polished condition compared to the as-received ones.
(2) The higher enrichment in aluminum and carbonate compounds observed by XPS on the coated AZ31 and AZ61 substrates in polished condition seems to be function of the treatment time and may be related with the different protective properties of the thin oxide/hydroxide film that spontaneously cover the surface of the alloy before the treatment.
(3) Combined analysis of XPS, EIS and hydrogen evolution data suggests a favorable effect for corrosion resistance of: (a) the amount of aluminum hydroxides and carbonate compounds observed on the external surface of the conversion coating, and (b) the absence of visible cracks or discontinuities on throughout the bulk of the conversion coating.
(4) The degree of improvement of the protection properties of the conversion coating formed on the AZ61 alloy induced by polishing the substrate seems to be related with the aggressiveness of the saline solutions. The carbonate enrichment has a favorable effect for corrosion resistance in weak or mild corrosive environments (0.006 M and 0.06 M NaCl solutions). In aggressive corrosive environment (0.6 M NaCl), a direct relationship has been observed between the enrichment of aluminum oxides and hydroxides and the improvement of the corrosion behavior.

## Figures and Tables

**Table 1.** Chemical composition of AZ31 and AZ61 alloys (wt%).

Alloy	Chemical Composition (wt%)						
	Al	Zn	Mn	Si	Fe	Ca	Mg
AZ31	3.1	0.73	0.25	0.02	0.005	0.0014	Balance
AZ61	6.2	0.74	0.23	0.04	0.004	0.0013	Balance
